# Effects of Field Simulated Marine Heatwaves on Sedimentary Organic Matter Quantity, Biochemical Composition, and Degradation Rates

**DOI:** 10.3390/biology11060841

**Published:** 2022-05-30

**Authors:** Santina Soru, Patrizia Stipcich, Giulia Ceccherelli, Claudia Ennas, Davide Moccia, Antonio Pusceddu

**Affiliations:** 1 Dipartimento di Scienze della Vita e dell’Ambiente, Università degli Studi di Cagliari, Via T. Fiorelli, 1, 09126 Cagliari, Italy; santina.soru@unica.it (S.S.); c.ennas@unica.it (C.E.); mocciadavide@unica.it (D.M.); 2 Dipartimento di Architettura, Design e Urbanistica, Università degli Studi di Sassari, Via Piandanna 4, 07100 Sassari, Italy; patriziastipcich@libero.it; 3 Dipartimento di Scienze Chimiche, Fisiche, Matematiche e Naturali, Università degli Studi di Sassari, Via Piandanna 4, 07100 Sassari, Italy; cecche@uniss.it

**Keywords:** marine heat waves, sedimentary organic matter, biopolymeric C, C degradation, ecosystem functioning

## Abstract

**Simple Summary:**

Marine heatwaves (MHWs) are intensifying due to global warming. Based on their effects on biochemical reactions, they are also likely to affect coastal biogeochemistry. We investigated organic matter quantity, composition and degradation rates in nearshore sediments affected by simulated MHWs, with 1.5 and 5.0 °C anomalies, before and after 3 and 11 weeks from the release of an artificial warm water plume. MHWs enhanced organic loads (by >100%), with larger effects in the short-term under the highest temperature anomaly. Phytopigment contents increased (by 50–90%) in the short term but decreased to initial values in the longer one. The autotrophic and lipid contents decreased with time (by 15–50% 53–79%, respectively), suggesting a drop in the nutritional quality of organic matter, along with a slowdown of its turnover. We contend that MHWs’ intensification will affect not only species and communities but will also alter sediment biogeochemistry and, possibly, the energy transfer towards higher trophic levels.

**Abstract:**

Since rising temperature (T) will enhance biochemical reactions and coastal marine sediments are hotspots of carbon cycling, marine heatwaves’ (MHWs’) intensification caused by climate change will affect coastal biogeochemistry. We investigated the effects of MHWs on sediment organic matter (OM) in a nearshore locality (NW Sardinia, Mediterranean Sea) receiving an artificial warm water plume generating T anomalies of 1.5–5.0 °C. Sediments were collected before and after 3 and 11 weeks from the initial plume release. Both MHWs influenced sedimentary OM quantity, composition, and degradation rates, with major effects associated with the highest T anomaly after 3 weeks. Both MHWs enhanced sedimentary OM contents, with larger effects associated with the highest T anomaly. Phytopigment contents increased in the short term but dropped to initial levels after 11 weeks, suggesting the occurrence of thermal adaptation or stress of microphytobenthos. In the longer term we observed a decrease in the nutritional quality of OM and a slowdown of its turnover mediated by extracellular enzymes, suggestive of a decreased ecosystem functioning. We anticipate that intensification of MHWs will affect benthic communities not only through direct effects on species tolerance but also by altering benthic biogeochemistry and the efficiency of energy transfer towards higher trophic levels.

## 1. Introduction

Anthropogenic global warming is rapidly emerging as a major threat to ecosystems worldwide [1,2]. Marine heatwaves (hereafter MHWs), as discrete but persistent (>5 days) positive (2–4 °C) anomalies in sea surface temperatures (SST) [3], are one of the most concerning and ubiquitous manifestations of global warming [4]. Marine warmth anomalies have become increasingly frequent in the last century [5], and episodes that have occurred across the last 20 years have caused severe biological, ecological, and economic consequences [6]. Recent projections indicate that such a surge in the frequency of MHWs could persevere for the whole current century [5] as the consequence of the persisting global ocean warming. Over the last two decades, several MHWs have been recorded globally [3,7], including in the Mediterranean Sea [8,9,10]. Being a miniature, shallow and warm ocean more prone to climate change than the open oceans [11], the annual mean SST of the Mediterranean basin is expected to increase from +1.5 °C to +3 °C by the end of the 21st century, fostering MHWs’ occurrence [9,12].

MHWs can have severe impacts on marine ecosystems [1,13,14,15,16,17]. Most of the knowledge accumulated regarding the effects of heatwaves on marine ecosystems derived from studies that assessed mass mortality, abundance reduction and changes in community structures of macro-and mega-benthos populations in situ [13,18,19,20,21,22,23,24]. Other studies carried out manipulative experiments on different marine communities and organisms to examine their response to warming [25,26,27,28,29]. Nevertheless, little research effort has been made to understand how temperature rise associated with MHWs could affect the nutrient and C cycling in coastal sediments [30,31], and to date, little, if any, attention has been given to the effects of MHWs on organic matter gross biochemical composition and degradation rates in coastal sediments. Since about half of annual marine C burial takes place in shallow water ecosystems [32], and temperature affects the rates of any (bio)chemical reaction, we predict that MHWs will affect C stocks and degradation rates in coastal marine ecosystems.

In order to provide new insights into this knowledge gap, we investigated short-term changes in quantity, biochemical composition, and degradation rates of organic matter in coastal sediments exposed to two simulated marine heatwaves, one simulating ocean temperatures corresponding to the future low emission scenario (MT) and one corresponding to a high emission scenario (HT) [33], generated by the cooling systems of an electric generation plant located in northwest Sardinia (Mediterranean Sea). More specifically, we tested the null hypothesis by which sedimentary organic matter quantity, biochemical composition (in terms of protein, carbohydrate, lipid and phytopigment contents), and potential degradation rates (expressed as rates of extracellular enzymatic degradation of proteins and carbohydrates) are not influenced by MHWs’ occurrence and intensity (in terms of temperature anomaly).

## 2. Materials and Methods

### 2.1. Study Area and Sampling Strategy

This study was conducted during the summer of 2020 in North-Western Sardinia (Italy, Western Mediterranean), in front of the Fiume Santo thermoelectric plant (40.85° N, 8.30° E), set up in the 1960s to supply electricity to Sardinia. The plant consists of two coal-fired units, each with a nominal power of 320 MW. To cool the whole system, seawater is continuously taken from the sea at 1 km from the coast and released back into the shore (12–24 m^3^ s^−1^) about 6–8 °C warmer (Power Plant Water, PPW). This creates a marked seawater temperature gradient in the vicinity of the discharge point which is conceivably moderated by season and weather conditions. The historical occurrence, duration, and intensity of MHWs occurring in the last 20 years in the study area have been characterized previously [29]. To run our experiment, we profited from ca. 5 weeks of inactivity of the power plant.

In order to evaluate the effects of the simulated MHWs on the sedimentary organic matter attributes, three sites were identified: (i) a “Control Temperature” site (CTRL), unaffected by PPW; (ii) a “Medium Temperature” site (MT) with PPW largely mixed with the natural seawater, with a mean surface T positive delta (compared to the control site) of ca. 2 °C; and a “High Temperature” site (HT) with PPW minimally mixed with the natural water, with a mean surface T positive delta (compared to the control site) of ca. 6 °C.

Sediment sampling was carried out on 14 July (T_0_, before the power plant ignition and the consequent injection of warm water at sea), 3 August (T_1_, 3 weeks after the beginning of warm water injection), and 1 October 2020 (T_2_, 11 weeks after the beginning of warm water injection). Samples collected after 3 and 11 weeks from the power plant ignition (T_1_ and T_2_, respectively) were assumed to represent medium- and long-term duration steps of the simulated MHVs.

Six independent replicated sediment cores were collected at ca. 3 m of depth at each station and sampling date using plexiglass corers (4.7 cm internal diameter) operated manually by SCUBA divers. The top first cm of each sediment core was stored in Petri dishes at −20 °C until analyses.

Temporal variations in sea bottom temperature (at ca. 3 m of depth) were similar at the three study sites, with a clear increase in T_1_ (after 3 weeks from the initial plume injection) and a partial recovery in T_2_ (after 11 weeks; Figure S1A). The simulated MHWs resembled either current or future scenarios according to Hobday et al. [3]: the magnitude of the two T anomalies corresponded to conditions observed in the study area during the 2000–2009 decade (+1.4–1.8 °C) and those expected in the future under the worst prediction scenarios (+4.5–5.1 °C) [29] (Figure S1B).

### 2.2. Biochemical Composition of Sediment Organic Matter

Chlorophyll-a and phaeopigment analyses were carried out according to Danovaro [34]. Pigments were extracted (overnight at 4 °C in the dark) from triplicate superficial (0–1 cm) 0.1 g sediment subsamples using 5 mL of 90% acetone as the extractant. Extracts were analyzed fluorometrically (430 nm excitation and 665 nm emission wavelengths) to estimate chlorophyll-a (Chla), and, after acidification with 200 μL 0.1N HCl, phaeo-pigment concentrations. Total phyto-pigment was defined as the sum of chlorophyll-a and phaeopigment concentrations and, once converted into carbon © equivalents using 40 μg C μg phytopigment^−1^ as a conversion factor, utilized to estimate the fraction of organic material of autotrophic origin [35]. Although the C:Chla can vary from 10 to 100 (on average 35 for phytoplankton), we used the conversion factor proposed in Pusceddu et al. [35] to allow comparison with other studies carried out in a variety of shallow coastal aquatic environments [36].

Protein, carbohydrate, and lipid contents were determined spectrophotometrically according to the protocols detailed in Danovaro [34]. More specifically, proteins were determined according to Lowry et al. [37], as modified by Hartree [38] and Rice [39], using the Folin-Ciocalteau reagent in a basic environment and expressed as bovine serum albumin equivalents. The procedure proposed by Gerchakov and Hatcher [40], based on the phenol and concentrated sulfuric acid reaction with saccharides, was used to determine carbohydrates, then expressed as D (+) Glucose equivalents. Lipids, after extraction in chloroform: methanol (1:1, vol:vol) [41], and evaporation in a dry hot bath at 80 to 100 °C for 20 min, were determined after the sulfuric acid carbonization procedure [42] and expressed as tripalmitin equivalents. For each biochemical assay, blanks were obtained using pre-calcinated sediments (450 °C for 4 h). Protein, carbohydrate, and lipid concentrations were converted into C equivalents using the conversion factors 0.49, 0.40, and 0.75 mgC mg^−1^, respectively, obtained from the C contents of the respective standard molecules (albumin, glucose and tripalmitin, respectively), and their sum was reported as the biopolymeric C (BPC) [43].

In order to assess the variations in the relative contribution of the basic organic matter’s biochemical components (C equivalents of protein, carbohydrate, lipid and phyto-pigment sedimentary contents) between the three temperature treatments, an index of biochemical diversity (IBD) was calculated as follows:

IBD = 1 − (b^2^_1_ + b^2^_2_ + b^2^_3_ + …b^2^_n_)

where b is the relative contribution of each biochemical compound (i.e., protein, carbohydrate, lipid and phytopigment carbon equivalents) to the cumulative sum of total biopolymeric carbon (BPC) and phytopigment C loads, and n is the number of biochemical compounds. Since IBD has a rank inversely related to biochemical homogeneity, we calculated the IBD-1 value; for n = 4 compounds, as in this study, the IBD-1 index ranges from 0 (minimum homogeneity) to 0.75 (maximum homogeneity).

### 2.3. Extracellular Enzymatic Activities, C Degradation Rates and Turnover

Organic matter degradation rates were estimated from aminopeptidase and β-glucosidase activities, determined by the cleavage of fluorogenic substrates (L-leucine-4-methylcoumarinyl-7-amide, for aminopeptidase; 4 methylumbelliferone-D-glucopyranoside, for β-glucosidase) at saturating concentrations [44]. Extracellular enzymatic activities were measured after the addition of 150 μL of substrate to 1 mL of a slurry prepared using 1:1 volume of filtered (0.2 μm) and sterile seawater and sediment (substrate final concentration 200 μM) [44]. Substrate incubations were performed in the dark at in situ temperature for 1 h. After incubation, the slurries were centrifuged (3000 rpm, 5 min) and supernatants were analyzed fluorometrically (at 365 nm excitation, 455 nm emission for β-glucosidase, and 380 nm excitation, 440 nm emission for aminopeptidase) [44]. Data were normalized to sediment dry weight (60 °C, 24 h) and reported as nanomole of substrate released per g of sediment dry weight h^−1^. Protease and glucosidase activities were converted into C equivalents using 72 as a conversion factor (estimated from the C content of the fluorescent component released after reaction with the enzymes) and their sum, reported as the potential C degradation rate (μgC g^−1^ h^−1^). The turnovers (per day) of the whole protein and carbohydrate pools were calculated as the ratios of the hourly C degradation rates (once multiplied by 24) and the whole protein and carbohydrate C contents in the sediment [45]. Although these estimates are only potential (maximum) rates of protein and carbohydrate turnover, they are considered good proxies of ecosystem functioning [45].

### 2.4. Effect Size

In order to visualize the magnitude of the reported effects on organic matter quantity, biochemical composition and diversity, and degradation rates in a standardized unambiguous metric regardless of the initial differences among sites, the forest plot representation was used based on the effect magnitude metric. The effect magnitude quantifies the results of an experiment as the log-proportional change between the mean (X) of treatment (T) and a control (C) group, as follows:

R_i_ = ln (XT_i_/XC_i_)

In this study, R_i_ is the log–response ratio for the variable i, and XT_i_ and XC_i_ are the mean values of the metric for the heated (MT or HT) and control (CTRL) sites, respectively.

### 2.5. Statistical Analyses

In order to test the null hypothesis by which variations in organic matter quantity, biochemical composition (in terms of protein, carbohydrate, lipid, and phytopigment contents), degradation rates and turn-over time among treatments and sampling times, permutational analyses of variance (PERMANOVA) [46] were carried out in either the uni- or multi-variate context with two fixed and orthogonal factors: treatment (Control “CTRL”, Medium Temperature “MT” and High Temperature “HT” anomaly) and time (T_0_, T_1_ and T_2_). PERMANOVA is a semiparametric method described as a geometric partitioning of multivariate variation in the space of a chosen dissimilarity measure according to a given ANOVA design, with *p*-values obtained using appropriate distribution-free permutation techniques. Since PERMANOVA on one response variable using Euclidean distance yields the classical univariate F statistic, PERMANOVA can also be used to perform univariate ANOVA, but where *p* values are obtained by permutation [47], thus avoiding the assumption of normality [48]. The analyses were carried out on Euclidean distance-based resemblance matrixes obtained from untransformed data, using 999 random permutations of the appropriate units. When significant differences were observed, pairwise tests were also carried out to ascertain patterns of differences among treatments and/or sampling times. Multivariate differences in organic matter biochemical composition (in terms of protein, carbohydrate, lipid and phytopigment contents) were visualized with a biplot after a canonical analysis of the principal coordinates (CAP). CAP allows identification of an axis through the multivariate cloud of points that is best at separating the groups. The motivation for the CAP routine arose as sometimes there are real differences among a priori groups in multivariate space that cannot be easily seen in an unconstrained ordination (as in PCA or MDS plots [49]). To quantify the homogeneity of dispersion among the data, a PERMDISP test was also carried out. Differences in the magnitude of the simulated heatwave effect among sampling times were determined after post-hoc pairwise tests. PERMANOVA, CAP and PERMDISP tests were carried out through the software PRIMER 6+, using the included routine package PERMANOVA [49].

## 3. Results

### 3.1. Effects of the Thermal Anomalies on Organic Matter Quantity and Biochemical Composition

Differences in organic matter quantity and biochemical composition largely depended on the interaction between treatments and sampling times (with exceptions for lipid and chlorophyll-a contents, and the autotrophic fraction of biopolymeric C; Table 1). Post-hoc tests were carried out to ascertain separately: (i) differences among treatments before (T_0_) and after (T_1_ and T_2_) PPW injection, and (ii) differences among sampling times in each treatment.

**Figure 1:**
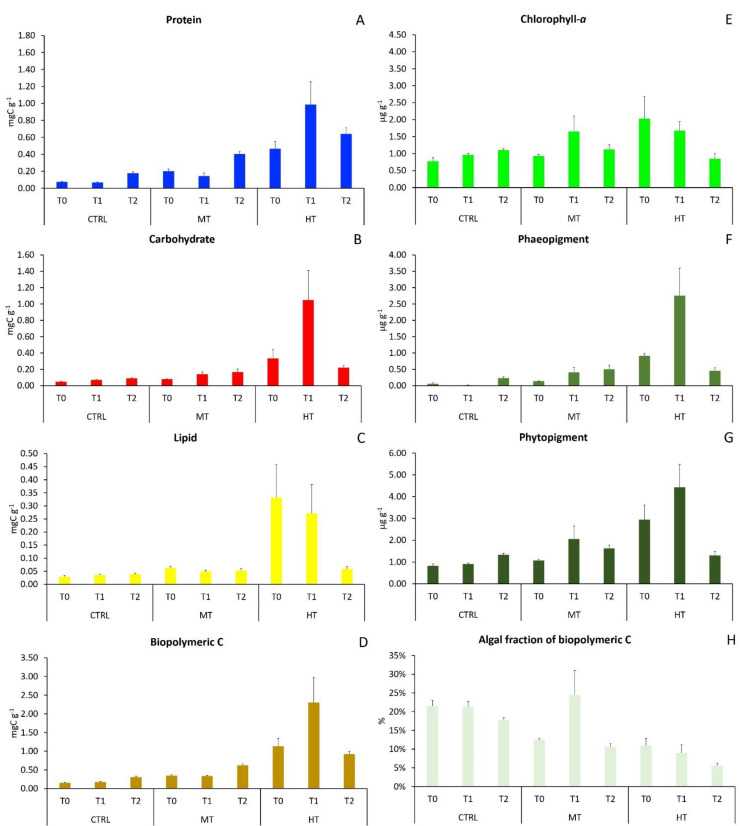
Changes in organic matter sedimentary contents in the three sampling sites at each sampling time: (**A**) protein, (**B**) carbohydrate, (**C**) lipid, (**D**) biopolymeric C, (**E**) chlorophyll-a, (**F**) phaeopigment, (**G**) total phytopigment, and (**H**) autotrophic fraction of biopolymeric C. CTRL = control; MT = medium temperature anomaly; HT = high temperature anomaly. T_0_ = before PPW injection; T_1_ = after 3 weeks from PPW injection; T_2_ = after 11 weeks from PPW injection. Error bars are standard errors (n = 6).

Before the injection of PPW, protein, carbohydrate, lipid, biopolymeric C and total phytopigment sedimentary contents differed significantly among treatments (Supplementary Table S1A), with much higher contents (up to seven times) in the HT site and lower in the CTRL (Figure 1A–G), whereas organic matter biochemical composition was rather similar among treatments (Supplementary Table S1A), with protein being the dominant class (40–58% of biopolymeric C), followed by lipids (36–58%) and carbohydrates. Quantitative differences in protein, carbohydrate, lipid and biopolymeric C contents among treatments were generally preserved in T_1_ (3 weeks after PPW injection) and T_2_ (11 weeks after), whereas differences in phytopigment contents weakened from T_1_ to T_2_ (Figure 1E–G). The autotrophic fraction of biopolymeric C, a proxy of sediment organic matter nutritional quality, was about two-fold higher in CTRL sediments than in MT and HT during the entire study period (Figure 1H).

**Figure 2:**
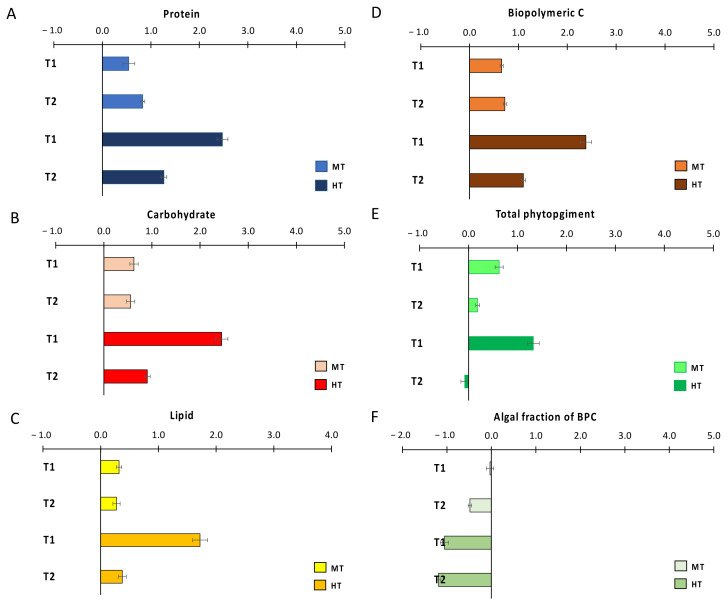
Size effects of temperature anomalies generated by the simulated heat wave on OM sedimentary contents: (**A**) protein, (**B**) carbohydrate, (**C**) lipid, (**D**) biopolymeric C, (**E**) total phytopigment, and (**F**) the autotrophic fraction of biopolymeric C. CTRL = control; MT = medium temperature anomaly; HT = high temperature anomaly. T_1_ = after 3 weeks from PPW injection; T_2_ = after 11 weeks from PPW injection. Error bars are standard errors (n = 6).

In order to visualize the effects of thermal anomalies on organic matter sedimentary contents, once having subtracted the differences among treatments at T_0_, we plotted the effect size for protein, carbohydrate, lipid, biopolymeric C, total phytopigment and autotrophic fraction of BPC against values in the CTRL treatment in T_1_ and T_2_ (Figure 2). After 3 weeks (T_1_), the injection of PPW determined a clear and significant increase in the sedimentary contents of all the investigated classes of organic compounds when compared to those in CTRL (Table 2), with effect sizes consistently larger in HT than in MT (Figure 2A–E). After 11 weeks from the initial PPW injection (T_2_), though organic matter contents in both MT and HT remained significantly higher than those in CTRL (Table 2), the positive effect of the thermal anomaly smothered this, when compared to that in T_1,_ for all classes of organic compounds, apart from phyto-pigments, whose contents returned close to those encountered in CTRL (Figure 2A–E). The effect of the thermal anomaly on the autotrophic fraction of biopolymeric C in MT was null in T_1_ and negative in T_2_, and negative in both T_1_ and T_2_ in HT; in both sampling times, the effect was consistently more negative in HT than in MT (Figure 2F).

**Figure 3:**
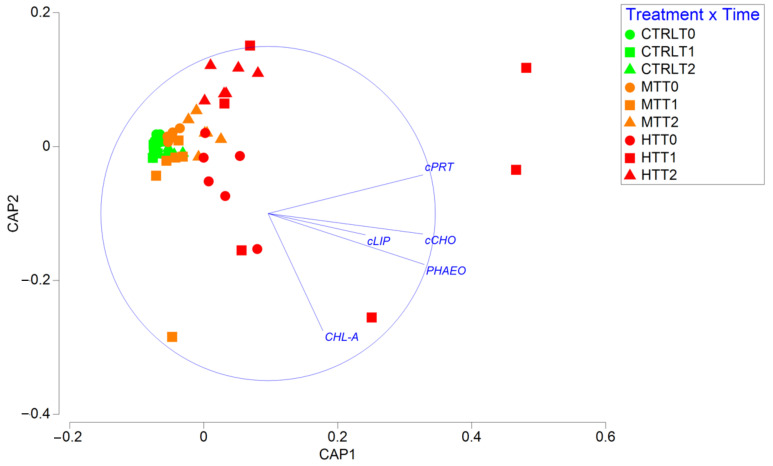
Biplot obtained after CAP analysis showing differences in the biochemical composition of sedimentary organic matter among treatments and sampling times. cPRT = protein; cCHO = carbohydrate; cLIP = lipid; Chl-a = chlorophyll-a; Phaeo = phaeo-pigment. CTRL = control; MT = intermediate temperature anomaly; HT = high temperature anomaly. T_0_ = before PPW injection; T_1_ = after 3 weeks from PPW injection; T_2_ = after 11 weeks from PPW injection.

The results of the two-way PERMANOVA test revealed a significant effect of the interaction between treatment and sampling time on organic matter biochemical composition (Table 1). The biplot made after the CAP analysis (Figure 3) reveals that organic matter biochemical composition in CTRL and MT remained relatively homogeneous during the entire study period.

Notably, organic matter composition in HT after 3 weeks from PPW injection was largely different from that in all other treatments and sampling times, then, in T_2_ (after 11 weeks from PPW injection) returned to resemble the composition observed in T_0_ and T_1_. As corroborated by the PERMDISP test, differences among replicates, a proxy for compositional heterogeneity, remained low in CTRL during the entire study period, whereas in both MT and HT it increased in T_1_ and then decreased again in T_2_ (Figure S2). Overall, the effect of the thermal anomaly on the biochemical diversity index was consistently negative in both T_1_ and T_2_, and increased with time in both treatments, indicating a progressive increase in organic matter heterogeneity (Figure S3A), mostly associated with an increase in the protein fraction at the expense of the lipid one (Figure S3B).

### 3.2. Sedimentary Organic Matter Degradation Rates

**Figure 4:**
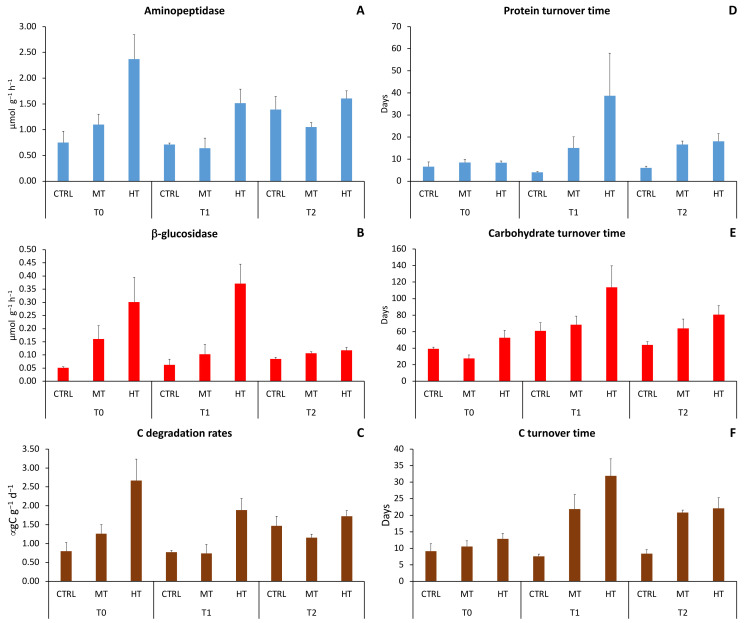
Changes in extracellular enzymatic (**A**) aminopeptidase, (**B**) β-glucosidase activities, (**C**) C degradation rates, (**D**) protein turnover time, (**E**) carbohydrate turnover time, and (**F**) C turnover time in the three sampling sites at each sampling time. CTRL = control; MT = medium temperature anomaly; HT = high temperature anomaly. T_0_ = before PPW injection; T_1_ = after 3 weeks from PPW injection; T_2_ = after 11 weeks from PPW injection.

Extracellular enzymatic activities were characterized by a significant effect of the simulated heatwaves but, with the exception of protein, carbohydrate and C turnover times, differences among treatments did not vary with time (Table 3; Figure 4A–C).

Before (T_0_) and after 3 weeks (T_1_) from PPW injection, aminopeptidase and β-glucosidase activities at HT were ca. two–three times higher than those in the MT and the control site. After 11 weeks from PPW injection (T_2_), such differences weakened (Supplementary Table S2A,B), with values in HT ca. 1–1.5 times higher than those in CTRL and MT. Aminopeptidase activity and C degradation rates remained constant between T_0_ and T_1_ and increased in T_2_ at CTRL, decreased in T_1_ and increased again in T_2_ at MT, and decreased in T_1_ and stabilized in T_2_ at HT. β-glucosidase activity slightly increased over time in CTRL, decreased in T_1_ and stabilized in T_2_ at MT, whereas in HT it remained constant between T_0_ and T_1_ and strongly decreased in T_2_. Turnover time of proteins, carbohydrates and C increased in both MT (ca. 1.5 times) and HT (ca. 8 times) in T_1_ then slightly recovered in T_2_ (Figure 4D–F).

**Figure 5:**
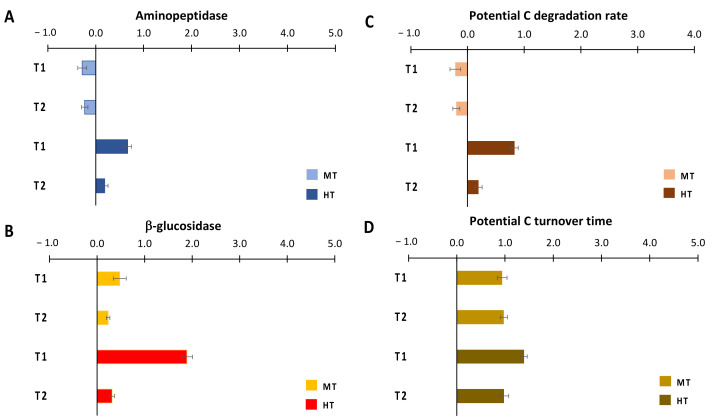
Size effects of temperature anomalies generated by the simulated heat wave on extracellular enzymatic (**A**) aminopeptidase and (**B**) β-glucosidase activities, on (**C**) C degradation rates and (**D**) C potential turnover time. MT = medium temperature anomaly; HT = high temperature anomaly. T_0_ = before PPW injection; T_1_ = after 3 weeks from PPW injection; T_2_ = after 11 weeks from PPW injection.

The effects of the temperature anomalies on aminopeptidase activities differed among treatments, being negative at MT and positive at HT (Figure 5A), whilst they were consistently positive on β-glucosidase activity in both treatments, much higher in T_1_ at HT than in all other cases (Figure 5B). Like aminopeptidase activity, C degradation rates were negatively affected by the simulated heatwave at MT and were stimulated at HT, with a size effect in T_1_ much higher than that in T_2_ (Figure 5C). C turnover time increased similarly in MT and HT (Figure 5D).

## 4. Discussion

### 4.1. MHWs Effects on Sedimentary Organic Matter Quantity, Biochemical Composition, and Nutritional Quality

Mean ocean surface temperature has increased by approximately 0.13 °C per decade over the past 100 years due to the massive heat adsorption by the oceans in response to the global warming caused by the increase of greenhouse gases in the atmosphere [50,51]. The consequences of oceans warming are multiple and affect both physical-chemical and biological features of the world’s oceans [30,52]. Evidence that global warming is leading to progressively more frequent and intense MHWs is accumulating [4,12], along with proofs of MHW impacts on marine species, habitats, and communities [21,22,53,54]. Nonetheless, the effects of MHWs on sedimentary organic matter contents, composition and degradation rates are, to the best of our knowledge, so far to be assessed.

Quantity and biochemical composition (in terms of proteins, carbohydrates, and lipids) of sedimentary organic matter are commonly used as proxies of the trophic state of coastal marine sediments [55,56]. While increasing biopolymeric C contents can be interpreted as an increase in the overall food availability for benthic consumers, variations in its biochemical composition and the autotrophic fraction of biopolymeric C influence its nutritional quality [36].

We show here that persistent MHWs (up to 11 weeks), irrespectively of the generated T anomaly, can lead to a consistent increase in sedimentary contents of all classes of organic compounds, when compared to the reference site uninfluenced by the MHW, with the highest T anomaly effect size larger than that of the intermediate one. This result would indicate that, at least in the short-term, MHWs can cause a localized increase in the whole amount of food for benthic consumers. We also report that, after 3 weeks from the initial release of the PPW plume, phytopigment sedimentary contents increased, irrespectively of the T anomaly level, but dropped down to levels observed before PPW injection after 11 weeks. While the positive response of phytopigments in the shorter term could be due to an increased microphytobenthos production stimulated by increased temperature range [57] and rising C incorporation rates [58], the prolonged exposition to the MHWs at week 11 could have caused a severe cellular stress. This hypothesis is corroborated by the observed positive effects of both MHWs on sedimentary lipid contents, which could have been caused by the increase in the lipid production of benthic microalgae in response to rising temperature and oligo-trophication [59]. Our hypothesis is also corroborated by previous studies showing that the effect of heatwaves on marine phytoplankton (and, thus, conceivably, on microphytobenthos) depends on the intensity of the heatwave [60], and that more intense heatwaves usually result in increased mortality [28]. These results, thus, suggest that, under a sort of negative feedback path, more persistent and more intense MHWs will not only cause a general oligo-trophication of the surface ocean (because of enhanced nutrient limitation), but will also impair the survivorship of microphytobenthos, thus ultimately impairing C sequestration processes in nearshore sediments. Moreover, in the longer term (at week 11), we observed a general decrease in the autotrophic fraction of biopolymeric C, again with an effect size caused by the highest T anomaly more negative than that caused by the lowest one. Phytopigments in shallow coastal sediments are a proxy of the amount of organic matter produced by photosynthesis [55] and their contribution to biopolymeric C is proportional to the bio-digestible (labile) fraction of biopolymeric C [36,61]. The observed decrease in the autotrophic fraction of biopolymeric C is indicative of a progressive depletion of sedimentary organic matter nutritional quality, which is also corroborated by the decrease in the high-energy lipid fraction of biopolymeric C along with a general progressive decrease in organic matter biochemical diversity. Thus, our results indicate that persistent and prolonged MHWs, besides their direct effects on benthic fauna and communities’ survivorship [62], could indirectly influence their ecological performance by altering the nutritional quality of the available food, with larger negative consequences associated with the most severe MHWs.

Recent modelling exercises showed that, at the end of the 21st century, a warmer Mediterranean Sea could be characterized by an overall expansion of P-limitation and a 10% reduction in phytoplankton net primary productivity [63], according to a predicted trophic attenuation of temperate seas with increasing sea temperature [64]. Conceivably, the ecological consequences of this climate change-related oligo-trophication of shallow areas of the Mediterranean Sea [65] could be locally exacerbated during prolonged events of MHWs. Although we have not investigated the responses of benthic fauna to the simulated MHWs, we could infer that, according to the optimal foraging theory [66], these persistent and prolonged events of thermal anomaly associated with MHWs could also affect the benthic community trophic structure, favoring species with high thermal tolerance and a preference for high quantities of nutritionally poor organic matter over species with low thermal tolerance and a preference for low quantities of nutritionally rich food. Moreover, our results confirm previous contentions, by which increasing frequency of more intense heatwaves could impair community resilience to withstand subsequent heatwaves [25,60,62].

### 4.2. MHWs’ Effects on Organic C Degradation Rates

Food availability for benthic consumers depends not only on organic matter quantity, biochemical composition, and nutritional quality, but also on the rates at which complex organic matter is made progressively more prone to consumer assimilation through microbial activities. This step is crucial for the degradation of marine sedimentary organic matter, which is generally dominated by large and relatively refractory polymeric molecules [36] and, thus, must undergo extracellular enzymatic hydrolysis to become nutritionally available for higher trophic levels. C degradation rates mediated by extracellular enzymes are influenced by temperature, so that rates of biogeochemical processes generally increase with increasing temperatures [67,68]. Studies investigating the effects of temperature on extracellular enzymes in marine sediments have generally dealt with seasonal and geographical variability patterns [69,70,71]. To the best of our knowledge, our study is the first ever providing insights on the effects of MHWs and associated T anomalies on C degradation rates mediated by extracellular enzymatic activities, which have been repeatedly used as a proxy of benthic ecosystem functioning [45,72].

We report here that, although both simulated MHWs caused an overall slowdown of C turnover (i.e., an increased C turnover time), the one generating a narrower T anomaly caused a different response of aminopeptidase and β-glucosidase activities, with the former depressed and the latter stimulated. Instead, the most severe MHW, associated with a larger T anomaly, determined a positive response of both activities, more relevant in the short-term, then attenuating in the long-term. These results indicate that the extent of the generated T anomaly is a crucial parameter of MHWs influencing differently the microbial-mediated C degradation. Nonetheless, our results suggest that MHWs, according to our initial hypothesis, can exert significant effects on the rates of C degradation, apparently enhancing ecosystem functioning. Heterotrophic microbes, through the microbial loop, are the most important nutrient flywheel in marine food webs [73]. According to the size–reactivity model, microbes selectively degrade high-molecular-weight molecules [74,75], as these compounds are generally too large to be transported across cell membranes [76]. Therefore, microbial extracellular enzymatic activity is the rate-limiting step in the degradation of organic matter in the oceans [77]. Our results, therefore, indicate that persistent MHWs, especially if generating T anomalies above 1.5 °C, can stimulate extracellular enzymatic activities and thus C degradation rates, causing a potential rise in the efficiency of energy transfer to higher trophic levels. This result agrees with the observed progressive decrease in the nutritional value of sedimentary organic matter and would suggest that MHWs can have severe effects on the whole trophic status of marine coastal sediments and, by cascade, on benthic trophic webs. However, the transfer of energy towards higher trophic levels is also a combination of changes in substrate quantity and rates of microbially mediated degradation. When combining the observed increase in substrate availability (proteins and carbohydrates) with the rise in their degradation rates mediated by enzymes under the larger T anomaly, we observed that, overall, the potential C turnover time increases, leading, ultimately, to a slowdown of benthic ecosystem functioning. This effect attenuates over time (i.e., at week 11 from PPW injection), possibly suggesting a sort of resilience of microbial activities, while the T anomaly persists. We cannot, however, exclude that this apparent recovery is due also to other mechanisms. For instance, the increase of total phytopigment contents at T1 under the largest T anomaly could have stimulated the degradation also of refractory buried C by self-priming [78] ultimately causing a decrease in biopolymeric C contents in the longer term.

## 5. Conclusions

The Mediterranean Sea, a semi-enclosed and relatively shallow basin, is one of the world regions most vulnerable to climate change [79], with a projected sea surface warming rate approximately 3–4 times higher than the global ocean ([80], and citations therein). Coastal aquatic ecosystems are among the most geochemically and biologically active areas of the biosphere and play a considerable role in the global biogeochemical cycles and, at the same time, they are among the most extensive and important carbon (C) reservoirs on the planet [81].

Our results indicate that benthic trophic status (in terms of organic matter quantity, composition, and nutritional quality) and ecosystem functioning (in terms of C degradation rates) of even very shallow nearshore marine sediments can be severely impaired by prolonged MHWs, with larger impacts associated with higher T anomalies. Based on these results, we can anticipate that the increase in frequency, intensity, and duration of MHWs, foreseen to cause abrupt ocean transitions in the coming decades [6,12] will cause not only direct effects on species and communities, hence overall threatening benthic biodiversity [18,21,80,82], but also provoke indirect effects by altering C biogeochemistry and the efficiency of energy transfer towards higher trophic levels.

## Figures and Tables

**Table 1:** Results of PERMANOVA testing for differences in sedimentary organic matter contents and biochemical composition among treatments (Tr: CTRL, MT and HT) and sampling times (Ti: T_0_, T_1_ and T_2_). Df = degrees of freedom; MS = mean square; F = statistic F; ** = *p* < 0.01; * = *p* < 0.05; ns = not significant. Reported is also the percentage of variation explained by each factor, their interaction and residual (Res; unexplained) variance. P (MC) = probability level after Monte Carlo simulations and 999 permutations.

Variable	Source	Df	MS	F	P (MC)	Explained Variance (%)
Protein	Tr	2	1.705	29.0	**	50.9
	Ti	2	0.149	2.5	ns	2.8
	Tr × Ti	4	0.205	3.5	*	13.6
	Res	45	0.058			32.6
Carbohydrate	Tr	2	1.149	11.8	**	26.1
	Ti	2	0.414	4.3	*	7.9
	Tr × Ti	4	0.403	4.2	**	22.7
	Res	45	0.097			43.3
Lipid	Tr	2	0.186	9.8	**	28.0
	Ti	2	0.040	2.1	ns	3.6
	Tr × Ti	4	0.042	2.2	ns	11.5
	Res	45	0.019			56.9
Chlorophyll-a	Tr	2	1.479	2.9	ns	7.6
	Ti	2	0.740	1.4	ns	1.8
	Tr × Ti	4	1.237	2.4	ns	17.1
	Res	45	0.516			73.5
Phaeopigment	Tr	2	8.211	16.231	**	28.4
	Ti	2	2.724	5.385	**	8.2
	Tr × Ti	4	3.216	6.358	**	29.9
	Res	45	0.506			33.5
Total phytopigment	Tr	2	16.554	12.5	**	27.3
	Ti	2	5.575	4.2	*	7.6
	Tr × Ti	4	5.499	4.2	**	22.5
	Res	45	1.322			42.6
Biopolymeric C	Tr	2	7.861	23.8	**	44.1
	Ti	2	0.794	2.4	ns	2.7
	Tr × Ti	4	1.372	4.2	**	18.3
	Res	45	0.330			34.8
Autotrophic fraction of biopolymeric C	Tr	2	0.064	16.9	**	37.1
	Ti	2	0.021	5.5	**	10.6
	Tr × Ti	4	0.009	2.5	ns	10.4
	Res	45	0.004			41.9
OM biochemical composition	Tr	2	12.731	10.6	**	24.2
	Ti	2	4.068	3.4	*	6.0
	Tr × Ti	4	5.103	4.3	**	24.6
	Res	45	1.196			45.2

**Table 2:** Results of the post-hoc tests assessing differences in the effect size of the thermal anomaly in MT and HT between pairs of sampling times. * = *p* < 0.05; ** = *p* < 0.01; *** = *p* < 0.001; ns = not significant.

Anomaly	Contrast	Protein	Carbohydrate	Lipid	Phytopigment	Biopolymeric C
MT	T_0_ vs. T_1_	**	ns	**	**	**
	T_0_ vs. T_2_	*	ns	**	*	*
	T_1_ vs. T_2_	*	ns	ns	**	ns
HT	T_0_ vs. T_1_	**	**	*	ns	**
	T_0_ vs. T_2_	**	***	***	***	***
	T_1_ vs. T_2_	***	***	***	***	***

**Table 3:** Results of PERMANOVA testing for differences in extracellular enzymatic activities, C degradation rates and turnover time among treatments (Tr: CTRL, MT and HT) and sampling times (Ti: T_0_, T_1_ and T_2_) Df = degrees of freedom; MS = mean square; F = statistic F; ** = *p* < 0.01; * = *p* < 0.05; ns = not significant. Reported is also the percentage of variation explained by each factor, their interaction and residual (Res; unexplained) variance. P (MC) = probability level after Monte Carlo simulations and 999 permutations.

Variable	Source	df	MS	F	P (MC)	% of Explained Variance
Aminopeptidase	Tr	2	4.760	13.7	**	35.1
	Ti	2	1.071	3.1	ns	5.7
	Tr × Ti	4	0.744	2.1	ns	9.5
	Res	45	0.347			49.7
β-glucosidase	Tr	2	0.184	14.7	**	34.6
	Ti	2	0.030	2.4	ns	3.6
	Tr × Ti	4	0.039	3.1	*	16.3
	Res	45	0.013			45.6
C degradation rate	Tr	2	6.744	15.0	**	38.1
	Ti	2	0.927	2.1	ns	2.9
	Tr × Ti	4	0.998	2.2	ns	9.9
	Res	45	0.451			49.1
Protein turnover time (d)	Tr	2	490.2	11.6	**	30.4
	Ti	2	178.7	4.2	*	9.2
	Tr × Ti	4	85.5	2.0	ns	8.8
	Res	45	42.3			51.6
Carbohydrate turnover time (d)	Tr	2	3842.8	6.9	**	18.2
	Ti	2	5574.0	10.0	**	27.8
	Tr × Ti	4	438.2	0.8	ns	0.0
	Res	45	559.7			54.0
C turnover time (d)	Tr	2	905.4	19.6	**	34.9
	Ti	2	424.1	9.2	**	15.3
	Tr × Ti	4	178.1	3.9	*	16.1
	Res	45	46.1			33.7

## Data Availability

The data presented in this study are available on reasonable request from the corresponding author.

## Supplementary Materials

The following supporting information can be downloaded at: https://www.mdpi.com/article/10.3390/biology11060841/s1. Supplementary Table S1A. Results of the pairwise comparisons testing for differences among treatments in sedimentary organic matter quantity and biochemical composition separately for each sampling time. Supplementary Table S1B. Results of the pairwise test comparison testing for differences in sedimentary organic matter quantity and composition between pairs of sampling time in each of the treatments. Supplementary Table S2A. Results of the pairwise tests assessing differences in extracellular enzymatic activities, protein, carbohydrate and C degradation rates and turnover times among treatments separately at each sampling time. Supplementary Table S2B. Results of the pairwise test comparison testing for differences in in extracellular enzymatic activities, protein, carbohydrate and C degradation rates and turnover times between pairs of sampling times separately for each treatment. Supplementary Figure S1. Temporal variations in (A) bottom temperature (°C) in the study sites and (B) temperature anomaly (°C) at the MT and HT sites after the injection of Power Plant Water (PPW). Supplementary Figure S2. Output of the of the homogeneity of dispersion analysis (PERMDISP) on (A) the sedimentary OM biochemical composition and (B) enzymatic activities among treatments and times. Supplementary Figure S3. Changes in the biochemical composition of sedimentary organic matter. (A) Size effects of temperature anomalies generated by the simulated heat wave on the index of biochemical diversity (IBD), and (B) changes in the relative (%) importance of protein, carbohydrate, and lipid contents in the biopolymeric C.
