# Supplementary material for: Effects of Field Simulated Marine Heatwaves on Sedimentary Organic Matter Quantity, Biochemical Composition, and Degradation Rates
Source: Biology (Basel). 2022 May 30;11(6):841. doi: 10.3390/biology11060841 (PMC9229934; doi:10.3390/biology11060841)
Supplement: Supplementary file 1 [file biology-11-00841-s001.zip › Supplementary Revised/Soru et al Supplementary Figure S2_amended2.pdf]

biology

MDPI

Article

Effects of Field Simulated Marine Heatwaves on Sedimentary Organic Matter Quantity, Biochemical Composition, and Degradation Rates

Santina Soru[1], Patrizia Stipcich[2], Giulia Ceccherelli[3], Claudia Ennas[1], Davide Moccia[1], Antonio Pusceddu[1]*

[1] Dipartimento di Scienze della Vita e dell'Ambiente, Università degli Studi di Cagliari, Via T. Fiorelli, 1, 09126 Cagliari, Italy; santina.soru@unica.it (S.S.); c.ennas@unica.it (C.E.); mocciadavide@unica.it (D.M.)
[2] Dipartimento di Architettura, Design e Urbanistica, Università degli Studi di Sassari, Via Piandanna 4, 07100 Sassari, Italy; patriziastipcich@libero.it
[3] Dipartimento di Scienze Chimiche, Fisiche, Matematiche e Naturali, Università degli Studi di Sassari, Via Piandanna 4, 07100 Sassari, Italy; cecche@uniss.it
* Correspondence: apusceddu@unica.it; Tel.: +39-070-6758053

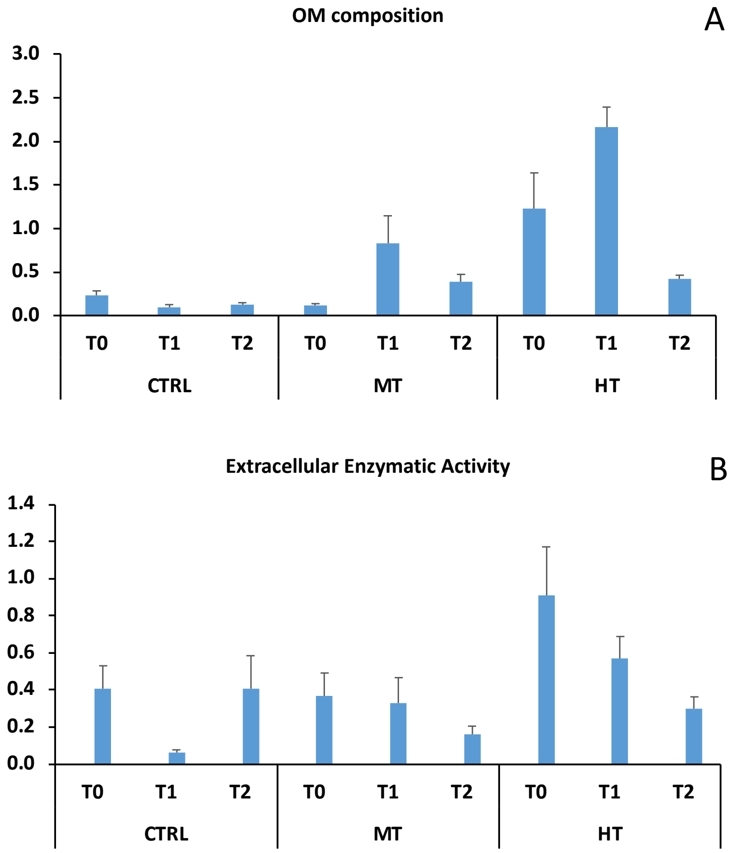

Supplementary Figure S2. Output of the of the homogeneity of dispersion analysis (PERMDISP) on (**A**) the sedimentary OM biochemical composition and (**B**) enzymatic activities among treatments and times.
