# Supplementary material for: Effects of Field Simulated Marine Heatwaves on Sedimentary Organic Matter Quantity, Biochemical Composition, and Degradation Rates
Source: Biology (Basel). 2022 May 30;11(6):841. doi: 10.3390/biology11060841 (PMC9229934; doi:10.3390/biology11060841)
Supplement: Supplementary file 1 [file biology-11-00841-s001.zip › Supplementary Revised/Soru et al Supplementary Table S1_amended2.pdf]

biology

Article

Effects of Field Simulated Marine Heatwaves on Sedimentary Organic Matter Quantity, Biochemical Composition, and Degradation Rates

Santina Soru[1], Patrizia Stipcich[2], Giulia Ceccherelli[3], Claudia Ennas[1], Davide Moccia[1], Antonio Pusceddu[1]*

[1] Dipartimento di Scienze della Vita e dell'Ambiente, Università degli Studi di Cagliari, Via T. Fiorelli, 1, 09126 Cagliari, Italy; santina.soru@unica.it (S.S.); c.ennas@unica.it (C.E.); mocciadavide@unica.it (D.M.)
[2] Dipartimento di Architettura, Design e Urbanistica, Università degli Studi di Sassari, Via Piandanna 4, 07100 Sassari, Italy; patriziastipcich@libero.it
[3] Dipartimento di Scienze Chimiche, Fisiche, Matematiche e Naturali, Università degli Studi di Sassari, Via Piandanna 4, 07100 Sassari, Italy; cecche@uniss.it
* Correspondence: apusceddu@unica.it; Tel.: +39-070-6758053

Supplementary Table S1A. Results of the pairwise comparisons testing for differences among treatments in sedimentary organic matter quantity and biochemical composition separately for each sampling time. CTRL = control; MT = medium anomaly; HT = highest anomaly. T_0 = before PPW injection; T_1 = 3 weeks after PPW injection; T_2 = 11 weeks after PPW injection. t = statistic t; p(MC) = probability level after Monte Carlo simulations; * = $p < 0.05$; ** = $p < 0.01$; *** = $p < 0.001$; ns = not significant.

Variable	Time	Contrast	t	P
Protein	T_0	CTRL vs. MT	5.237	**
		CTRL vs. HT	4.426	**
		MT vs. HT	2.876	*
	T_1	CTRL vs. MT	2.148	ns
		CTRL vs. HT	3.424	*
		MT vs. HT	3.111	*
	T_2	CTRL vs. MT	6.781	**
		CTRL vs. HT	6.205	**
		MT vs. HT	2.996	*
Carbohydrate	T_0	CTRL vs. MT	5.976	**
		CTRL vs. HT	2.635	*
		MT vs. HT	2.327	*
	T_1	CTRL vs. MT	2.421	*
		CTRL vs. HT	2.703	*
		MT vs. HT	2.497	*
	T_2	CTRL vs. MT	2.108	**
		CTRL vs. HT	4.585	**
		MT vs. HT	1.216	*
Lipid	T_0	CTRL vs. MT	3.124	*
		CTRL vs. HT	2.402	*
		MT vs. HT	2.149	ns
	T_1	CTRL vs. MT	1.937	ns
		CTRL vs. HT	2.090	ns
		MT vs. HT	1.998	ns
		CTRL vs. MT	2.200	ns

	T_2	CTRL vs. HT	2.320	*
		MT vs. HT	0.287	ns
Chlorophyll-*a*	T_0	CTRL vs. MT	1.319	ns
		CTRL vs. HT	1.899	ns
		MT vs. HT	1.676	ns
	T_1	CTRL vs. MT	1.491	ns
		CTRL vs. HT	2.646	*
		MT vs. HT	0.047	ns
	T_2	CTRL vs. MT	0.135	ns
		CTRL vs. HT	1.598	ns
		MT vs. HT	1.312	ns
Phaeopigment	T_0	CTRL vs. MT	2.361	*
		CTRL vs. HT	1.809	ns
		MT vs. HT	1.728	ns
	T_1	CTRL vs. MT	2.661	*
		CTRL vs. HT	3.268	*
		MT vs. HT	2.748	*
	T_2	CTRL vs. MT	2.130	ns
		CTRL vs. HT	2.272	*
		MT vs. HT	0.311	ns
Total phytopigment	T_0	CTRL vs. MT	2.479	*
		CTRL vs. HT	1.876	ns
		MT vs. HT	1.734	ns
	T_1	CTRL vs. MT	1.940	ns
		CTRL vs. HT	3.338	**
		MT vs. HT	1.963	ns
	T_2	CTRL vs. MT	1.732	ns
		CTRL vs. HT	0.142	ns
		MT vs. HT	1.341	ns
Algal fraction of biopolymeric C	T_0	CTRL vs. MT	5.692	**
		CTRL vs. HT	2.324	ns
		MT vs. HT	0.150	ns
	T_1	CTRL vs. MT	0.450	ns
		CTRL vs. HT	4.700	**
		MT vs. HT	2.267	*
	T_2	CTRL vs. MT	5.662	**
		CTRL vs. HT	13.027	**
		MT vs. HT	4.045	**
Biopolymeric C	T_0	CTRL vs. MT	7.940	**
		CTRL vs. HT	4.614	**
		MT vs. HT	3.693	**
	T_1	CTRL vs. MT	8.552	**
		CTRL vs. HT	3.200	*
		MT vs. HT	2.959	*
	T_2	CTRL vs. MT	7.494	**
		CTRL vs. HT	7.990	**
		MT vs. HT	3.678	**
	T_0	CTRL vs. MT	1.755	ns
		CTRL vs. HT	1.870	ns

OM biochemical composition		MT vs. HT	1.732	ns
	T_1	CTRL vs. MT	1.646	ns
		CTRL vs. HT	3.156	*
		MT vs. HT	2.401	*
	T_2	CTRL vs. MT	1.807	*
		CTRL vs. HT	2.883	**
		MT vs. HT	1.345	ns

Supplementary Table S1B. Results of the pairwise test comparison testing for differences in sedimentary organic matter quantity and composition between pairs of sampling time in each of the treatments. CTRL = control; MT = medium anomaly; HT = highest anomaly. T_0 = before PPW injection; T_1 = 3 weeks after PPW injection; T_2 = 11 weeks after PPW injection. t = statistic t; p(MC) = probability level after Monte Carlo simulations; * = $p < 0.05$; ** = $p < 0.01$; *** = $p < 0.001$; ns = not significant.

Variable	Term	Groups	t	P
Protein	CTRL	T_0 vs. T_1	0.785	ns
		T_0 vs. T_2	5.805	**
		T_1 vs. T_2	6.160	**
	MT	T_0 vs. T_1	1.323	ns
		T_0 vs. T_2	5.465	**
		T_1 vs. T_2	5.724	**
	HT	T_0 vs. T_1	1.848	ns
		T_0 vs. T_2	1.533	ns
		T_1 vs. T_2	1.248	ns
Carbohydrate	CTRL	T_0 vs. T_1	2.236	ns
		T_0 vs. T_2	3.558	*
		T_1 vs. T_2	1.534	ns
	MT	T_0 vs. T_1	2.028	ns
		T_0 vs. T_2	2.315	*
		T_1 vs. T_2	0.544	ns
	HT	T_0 vs. T_1	1.894	ns
		T_0 vs. T_2	1.007	ns
		T_1 vs. T_2	2.278	*
Lipid	CTRL	T_0 vs. T_1	1.784	ns
		T_0 vs. T_2	1.213	ns
		T_1 vs. T_2	0.791	ns
	MT	T_0 vs. T_1	1.074	ns
		T_0 vs. T_2	0.447	ns
		T_1 vs. T_2	0.565	ns
	HT	T_0 vs. T_1	0.368	ns
		T_0 vs. T_2	2.161	ns
		T_1 vs. T_2	1.918	ns
Chlorophyll-*a*	CTRL	T_0 vs. T_1	1.570	ns
		T_0 vs. T_2	2.825	*
		T_1 vs. T_2	2.188	*
	MT	T_0 vs. T_1	1.556	ns
		T_0 vs. T_2	1.271	ns
		T_1 vs. T_2	1.097	ns
	HT	T_0 vs. T_1	0.503	ns
		T_0 vs. T_2	1.760	ns
		T_1 vs. T_2	2.697	*
Phaeopigment	CTRL	T_0 vs. T_1	1.206	ns
		T_0 vs. T_2	3.229	**
		T_1 vs. T_2	5.182	**
	MT	T_0 vs. T_1	1.787	ns
		T_0 vs. T_2	3.010	*
		T_1 vs. T_2	0.494	ns

	HT	T_0 vs. T_1	0.582	ns
		T_0 vs. T_2	1.423	ns
		T_1 vs. T_2	2.718	*
Total phytopigment	CTRL	T_0 vs. T_1	0.845	ns
		T_0 vs. T_2	4.382	**
		T_1 vs. T_2	4.785	**
	MT	T_0 vs. T_1	1.670	ns
		T_0 vs. T_2	3.520	*
		T_1 vs. T_2	0.714	ns
	HT	T_0 vs. T_1	0.217	ns
		T_0 vs. T_2	1.587	ns
		T_1 vs. T_2	2.923	*
Algal fraction of biopolymeric C	CTRL	T_0 vs. T_1	0.076	ns
		T_0 vs. T_2	2.137	ns
		T_1 vs. T_2	2.042	ns
	MT	T_0 vs. T_1	1.822	ns
		T_0 vs. T_2	1.801	ns
		T_1 vs. T_2	2.106	ns
	HT	T_0 vs. T_1	1.052	ns
		T_0 vs. T_2	2.181	ns
		T_1 vs. T_2	1.415	ns
Biopolymeric C	CTRL	T_0 vs. T_1	1.320	ns
		T_0 vs. T_2	5.492	**
		T_1 vs. T_2	4.531	**
	MT	T_0 vs. T_1	0.388	ns
		T_0 vs. T_2	6.907	**
		T_1 vs. T_2	7.893	**
	HT	T_0 vs. T_1	1.681	ns
		T_0 vs. T_2	0.939	ns
		T_1 vs. T_2	2.068	ns
OM biochemical composition	CTRL	T_0 vs. T_1	1.545	ns
		T_0 vs. T_2	2.985	**
		T_1 vs. T_2	3.552	**
	MT	T_0 vs. T_1	1.579	ns
		T_0 vs. T_2	2.329	*
		T_1 vs. T_2	1.144	ns
	HT	T_0 vs. T_1	0.771	ns
		T_0 vs. T_2	1.532	ns
		T_1 vs. T_2	2.572	*
