# Supplementary material for: Effects of Field Simulated Marine Heatwaves on Sedimentary Organic Matter Quantity, Biochemical Composition, and Degradation Rates
Source: Biology (Basel). 2022 May 30;11(6):841. doi: 10.3390/biology11060841 (PMC9229934; doi:10.3390/biology11060841)
Supplement: Supplementary file 1 [file biology-11-00841-s001.zip › Supplementary Revised/Soru et al Supplementary Table S2_amended 2.pdf]

biology MDPI

Article

Effects of Field Simulated Marine Heatwaves on Sedimentary Organic Matter Quantity, Biochemical Composition, and Degradation Rates

Santina Soru[1], Patrizia Stipcich[2], Giulia Ceccherelli[3], Claudia Ennas[1], Davide Moccia[1], Antonio Pusceddu[1]*

[1] Dipartimento di Scienze della Vita e dell'Ambiente, Università degli Studi di Cagliari, Via T. Fiorelli, 1, 09126 Cagliari, Italy; santina.soru@unica.it (S.S.); c.ennas@unica.it (C.E.); mocciadavide@unica.it (D.M.)
[2] Dipartimento di Architettura, Design e Urbanistica, Università degli Studi di Sassari, Via Piandanna 4, 07100 Sassari, Italy; patriziastipcich@libero.it
[3] Dipartimento di Scienze Chimiche, Fisiche, Matematiche e Naturali, Università degli Studi di Sassari, Via Piandanna 4, 07100 Sassari, Italy; cecche@uniss.it
* Correspondence: apusceddu@unica.it; Tel.: +39-070-6758053

Supplementary Table S2A. Results of the pairwise tests assessing differences in evs.tracellular enzymatic activities, protein, carbohydrate and C degradation rates and turnover times among treatments separately at each sampling time. CTRL = control; MT = medium anomaly; HT = highest anomaly. T_0 = before PPW injection; T_1 = 3 weeks after PPW injection; T_2 = 11 weeks after PPW injection. t = statistic t; P(MC) = probability level after Monte Carlo simulations; * = $p < 0.05$; ** = $p < 0.01$; *** = $p < 0.001$; ns = not significant.

Variable	Term	Groups	t	P(MC)
Aminopeptidase	T_0	CTRL vs. MT	1.180	ns
		CTRL vs. HT	3.092	*
		MT vs. HT	2.464	*
	T_1	CTRL vs. MT	0.369	ns
		CTRL vs. HT	2.960	**
		MT vs. HT	2.618	*
	T_2	CTRL vs. MT	1.251	ns
		CTRL vs. HT	0.740	ns
		MT vs. HT	3.252	**
β-glucosidase	T_0	CTRL vs. MT	2.186	ns
		CTRL vs. HT	2.656	*
		MT vs. HT	1.313	ns
	T_1	CTRL vs. MT	0.925	ns
		CTRL vs. HT	4.016	**
		MT vs. HT	3.241	*
	T_2	CTRL vs. MT	2.591	*
		CTRL vs. HT	2.524	*
		MT vs. HT	0.777	ns
C degradation rate	T_0	CTRL vs. MT	1.389	ns
		CTRL vs. HT	3.074	**
		MT vs. HT	2.287	*
	T_1	CTRL vs. MT	0.133	ns

		CTRL vs. HT	3.602	**
		MT vs. HT	2.970	*
		CTRL vs. MT	1.178	ns
	T_2	CTRL vs. HT	0.847	ns
		MT vs. HT	3.206	*
Potential protein turnover time (d)	T_0	CTRL vs. MT	0.740	ns
		CTRL vs. HT	0.834	ns
		MT vs. HT	0.043	ns
	T_1	CTRL vs. MT	2.204	*
		CTRL vs. HT	3.879	**
		MT vs. HT	0.735	ns
	T_2	CTRL vs. MT	5.801	**
		CTRL vs. HT	3.428	**
		MT vs. HT	0.403	ns
Potential carbohydrate turnover time (d)	T_0	CTRL vs. MT	2.512	*
		CTRL vs. HT	1.427	ns
		MT vs. HT	2.487	*
	T_1	CTRL vs. MT	0.525	ns
		CTRL vs. HT	1.691	ns
		MT vs. HT	1.285	ns
	T_2	CTRL vs. MT	5.801	**
		CTRL vs. HT	3.428	**
		MT vs. HT	0.403	ns
C turnover time (d)	T_0	CTRL vs. MT	0.509	ns
		CTRL vs. HT	1.330	ns
		MT vs. HT	0.951	ns
	T_1	CTRL vs. MT	3.207	*
		CTRL vs. HT	4.686	*
		MT vs. HT	1.486	ns
	T_2	CTRL vs. MT	9.089	**
		CTRL vs. HT	4.005	**
		MT vs. HT	0.394	ns

Supplementary Table S2B. Results of the pairwise test comparison testing for differences in in evs.tracellular enzymatic activities, protein, carbohydrate and C degradation rates and turnover times between pairs of sampling times separately for each treatment. CTRL = control; MT = intermediate anomaly; HT = high anomaly. T_0 = before PPW injection; T_1 = after 3 weeks from PPW injection; T_2= after 11 weeks from PPW injection. t = statistic t; p(MC) = probability level after Monte Carlo simulations; * = $p < 0.05$; ** = $p < 0.01$; *** = $p < 0.001$; ns = not significant.

Variable	Term	Groups	t	P (MC)	P
Aminopeptidase	CTRL	T_0 vs. T_1	0.158	0.884	ns
		T_0 vs. T_2	1.894	0.091	ns
		T_1 vs. T_2	2.626	0.032	*
	MT	T_0 vs. T_1	1.635	0.115	ns
		T_0 vs. T_2	0.223	0.831	ns
		T_1 vs. T_2	1.906	0.081	ns
	HT	T_0 vs. T_1	1.557	0.145	ns
		T_0 vs. T_2	1.533	0.146	ns
		T_1 vs. T_2	0.287	0.797	ns
β-glucosidase	CTRL	T_0 vs. T_1	0.610	0.542	ns
		T_0 vs. T_2	4.583	0.004	**
		T_1 vs. T_2	0.960	0.351	ns
	MT	T_0 vs. T_1	0.905	0.407	ns
		T_0 vs. T_2	1.022	0.361	ns
		T_1 vs. T_2	0.132	0.894	ns
	HT	T_0 vs. T_1	0.587	0.588	ns
		T_0 vs. T_2	1.918	0.086	ns
		T_1 vs. T_2	3.395	0.014	*
C degradation rate	CTRL	T_0 vs. T_1	0.110	0.914	ns
		T_0 vs. T_2	1.994	0.073	ns
		T_1 vs. T_2	2.719	0.024	*
	MT	T_0 vs. T_1	1.524	0.152	ns
		T_0 vs. T_2	0.396	0.691	ns
		T_1 vs. T_2	1.644	0.120	ns
	HT	T_0 vs. T_1	1.212	0.244	ns
		T_0 vs. T_2	1.616	0.139	ns
		T_1 vs. T_2	0.487	0.637	ns
Potential protein turnover time (d)	CTRL	T_0 vs. T_1	1.265	0.241	ns
		T_0 vs. T_2	0.284	0.799	ns
		T_1 vs. T_2	2.243	0.049	*
	MT	T_0 vs. T_1	1.265	0.236	ns
		T_0 vs. T_2	3.685	0.004	*
		T_1 vs. T_2	0.285	0.779	ns
	HT	T_0 vs. T_1	2.772	0.020	*
		T_0 vs. T_2	2.768	0.024	*
		T_1 vs. T_2	0.320	0.759	ns

Potential carbohydrate turnover time (d)	CTRL	T_0 vs. T_1	2.157	0.072	ns
		T_0 vs. T_2	1.049	0.338	ns
		T_1 vs. T_2	1.607	0.152	ns
	MT	T_0 vs. T_1	3.658	0.004	*
		T_0 vs. T_2	3.008	0.011	*
		T_1 vs. T_2	0.296	0.780	ns
	HT	T_0 vs. T_1	2.173	0.058	ns
		T_0 vs. T_2	2.008	0.071	ns
		T_1 vs. T_2	0.657	0.530	ns
C turnover time (d)	CTRL	T_0 vs. T_1	0.698	0.507	ns
		T_0 vs. T_2	0.300	0.765	ns
		T_1 vs. T_2	0.656	0.544	ns
	MT	T_0 vs. T_1	2.383	0.035	*
		T_0 vs. T_2	5.501	0.001	**
		T_1 vs. T_2	0.231	0.821	ns
	HT	T_0 vs. T_1	3.518	0.004	**
		T_0 vs. T_2	2.551	0.022	*
		T_1 vs. T_2	1.612	0.146	ns
